# Reporting signals of selected extrapyramidal adverse events with four newer second-generation antipsychotics: a disproportionality analysis of FAERS spontaneous reports

**DOI:** 10.3389/fphar.2026.1921943

**Published:** 2026-10-12

**Authors:** Pan Ma, Qin Xiao, Yunpei Zhao, Xin Zhang, Huan Ma, Dan Chen, Linli Xie

**Affiliations:** 1 Department of Pharmacy, The First Affiliated Hospital of Army Medical University, Chongqing, China; 2 Department of Pharmacy, Shengjing Hospital, China Medical University, Shenyang, China

**Keywords:** disproportionality analysis, extrapyramidal symptoms, FAERS, pharmacovigilance, second-generation antipsychotics

## Abstract

**Background:**

Newer second-generation antipsychotics (SGAs) differ in pharmacology and approved indications, and extrapyramidal symptoms (EPS) remain clinically important. We investigated whether lurasidone, brexpiprazole, cariprazine, and lumateperone showed disproportionate reporting of selected EPS in the FDA Adverse Event Reporting System (FAERS).

**Methods:**

We conducted a retrospective pharmacovigilance signal-detection study of spontaneous reports using OpenVigil 2.1-MedDRA-v24 (FAERS Q1 2004-Q3 2025). Analyses were restricted to reports in which the target drug was the primary suspect. Narrow MedDRA sub-SMQs for Akathisia, Dyskinesia, and Dystonia and their Preferred Terms (PTs) were evaluated with the reporting odds ratio (ROR), proportional reporting ratio (PRR), and Bayesian information component (IC). Sensitivity analyses used a common calendar window (Q1 2020-Q3 2025) and the first 24 post-approval quarters for each drug.

**Results:**

All four drugs had positive reporting signals for the three selected outcomes, and Akathisia had the largest within-drug IC025. Drug-specific PT patterns included akathisia/restlessness, tardive dyskinesia, and focal dystonia terms. The four separately calculated drug-versus-database estimates were not tested against one another and are not comparative risk estimates. All 24 estimates in the two time-window sensitivity analyses retained a lower ROR confidence bound above one and IC025 above 0, although signal magnitudes varied across windows.

**Conclusion:**

The findings generate pharmacovigilance hypotheses for selected EPS only; they do not estimate incidence, comparative risk, or causality and should not guide preferential prescribing. Although positive signals persisted in both time-window sensitivity analyses, variation in signal magnitude, reporting behavior, differences in indication and concomitant treatment, the unexplained denominator discrepancy, and omission of Parkinson-like events require cautious interpretation and independent validation.

## Graphical Abstract

**Figure:**
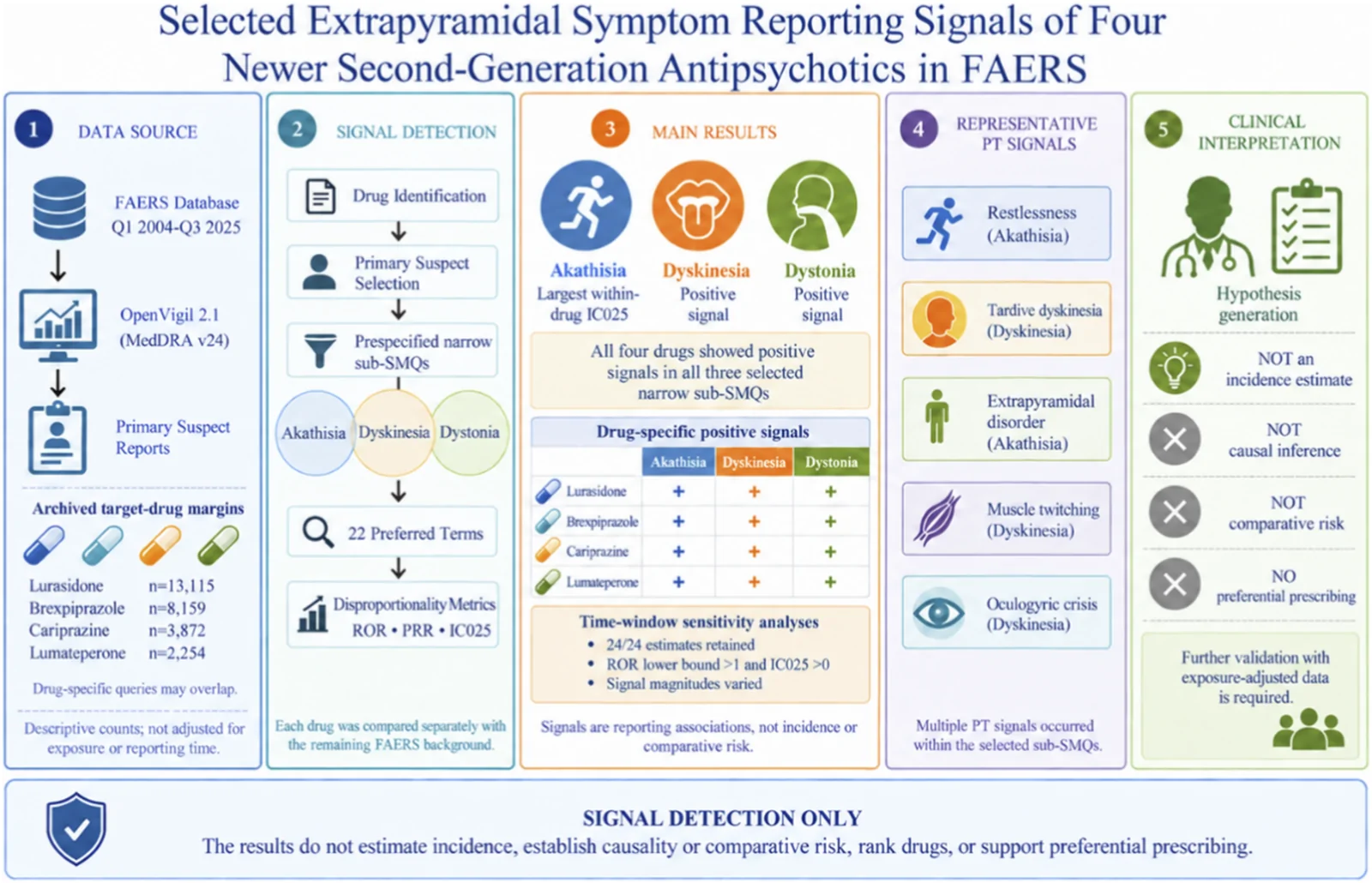

## 1 Introduction

Extrapyramidal symptoms (EPS) are clinically important movement disorders associated with antipsychotic treatment. Akathisia, dyskinesia, dystonia, and parkinsonian manifestations can impair functioning, reduce adherence, and complicate long-term care (Stahl et al., 2021; Siafis et al., 2023). Although second-generation antipsychotics (SGAs) generally produce fewer EPS than first-generation agents, clinically relevant EPS remain possible and vary with dose, receptor pharmacology, patient susceptibility, treatment duration, and concomitant therapy (Huhn et al., 2019; Siafis et al., 2023).

Lurasidone, brexpiprazole, cariprazine, and lumateperone are newer SGAs with distinct pharmacologic profiles and approved uses. Lurasidone is an antagonist at dopamine D2 and serotonin 5-HT2A/5-HT7 receptors and a partial agonist at 5-HT1A receptors. Brexpiprazole and cariprazine are D2/D3 partial agonists; cariprazine preferentially binds D3 receptors and has active metabolites with prolonged exposure. Lumateperone modulates serotonin, dopamine, and glutamate signaling (Corponi et al., 2019; de Bartolomeis et al., 2022; Pahwa et al., 2021). These differences provide a pharmacologic rationale for examining post-marketing movement-disorder reporting patterns, while not assuming that spontaneous reports measure comparative clinical risk.

Clinical trials and network meta-analyses characterize short-term tolerability in selected populations, whereas spontaneous-reporting systems can generate hypotheses about uncommon or delayed events after broader use (Huhn et al., 2019; Siafis et al., 2023). Drug-specific FAERS analyses have been reported for each of the four agents (Jiang et al., 2024; Zhao et al., 2024; Zhu et al., 2024; Luo et al., 2025), but they used drug-specific time windows and analytic specifications. The incremental contribution of the present study is the use of one OpenVigil snapshot, PS-only report selection, the same MedDRA v24 narrow sub-SMQ/PT mapping, and identical ROR/PRR/IC thresholds for all four agents. This harmonization supports consistent drug-specific signal characterization, not head-to-head safety comparison. FAERS lacks a valid exposure denominator; therefore, disproportionality estimates indicate reporting patterns and cannot establish incidence, causal effects, or comparative clinical risk (Cutroneo et al., 2024).

Accordingly, the clinical pharmacovigilance question was: which selected EPS categories and PTs are reported disproportionately for each of these four newer SGAs relative to the background of other reports in FAERS? We evaluated the narrow MedDRA sub-SMQs Akathisia, Dyskinesia, and Dystonia and their component PTs. The objective was signal detection and hypothesis generation, not estimation of comparative risk or prescribing preference.

## 2 Methods

### 2.1 Data source and preprocessing

FAERS is a spontaneous-reporting system that receives voluntary and mandatory safety reports. It is subject to under-reporting, stimulated and selective reporting, incomplete information, variable data quality, and the absence of a valid exposure denominator. Accordingly, the present retrospective analysis was designed for signal detection rather than estimation of incidence or causality (Cutroneo et al., 2024).

We queried the OpenVigil 2.1 online interface using its MedDRA-v24 installation and the FAERS snapshot covering Q1 2004 through Q3 2025 (Böhm et al., 2016). The online installation and snapshot were verified during revision on 1 August 2026; the exact calendar date of the original query was not retained in the analysis log and is reported here transparently. OpenVigil harmonizes drug and event fields and deduplicates follow-up versions by retaining one representative individual safety report per FAERS case (the most recent ISR for a CASEID). The unit of analysis was therefore a deduplicated safety report. Residual duplicates that cannot be recognized from case identifiers remain possible.

### 2.2 Drug identification and report selection

Drug records were identified separately using generic and common brand names (lurasidone/Latuda, brexpiprazole/Rexulti, cariprazine/Vraylar, and lumateperone/Caplyta), with product names normalized to the active substance in OpenVigil. We retained reports in which the target drug was coded as the primary suspect (PS). The four drug queries were not forced to be mutually exclusive; a report listing more than one study drug as PS could contribute to each relevant drug-specific query. For each drug–event analysis, the comparator comprised all other eligible FAERS reports in the same OpenVigil snapshot; reports involving the other study antipsychotics were not removed from that database background. Indications, concomitant medications, demographics, reporter country, outcomes, and quarterly counts were summarized when available.

### 2.3 Outcome definition

Events were coded with MedDRA version 24. We used narrow terms from three official hierarchical sub-SMQs within Extrapyramidal syndrome (SMQ code 20000095): Akathisia (20,000,096), Dyskinesia (20,000,097), and Dystonia (20,000,098) (MedDRA MSSO, 2021). Tardive dyskinesia was classified under Dyskinesia. A report could contribute more than one PT and more than one sub-SMQ when multiple coded events were present; overlapping PT labels were listed once in the 22-PT analysis set. The complete PT mapping is provided in Supplementary Table S1. Parkinson-like events (20,000,099) were not part of the locked prespecified extraction. Although the retained SMQ workbook contained aggregate Parkinson-like rows for lurasidone, brexpiprazole, and cariprazine, it contained no corresponding lumateperone 2 × 2 output; an analyzable four-drug set was therefore unavailable. We did not combine this incomplete *post hoc* output. Consequently, the study does not characterize the full EPS spectrum, and no inference about total EPS burden should be drawn.

To examine sensitivity to unequal surveillance duration and time since U.S. approval, we conducted two analyses for the primary sub-SMQs: (1) a common calendar window restricted to Q1 2020-Q3 2025 for all four drugs and (2) a product-age aligned window restricted to the first 24 post-approval quarters for each drug (Supplementary Table S3). In each analysis, the PS restriction, outcome definitions, disproportionality measures, thresholds, and drug-specific comparator design were unchanged, and both target-drug reports and the remaining-database background were restricted to the relevant window. The 24-quarter windows were Q4 2010-Q3 2016 for lurasidone, Q3 2015-Q2 2021 for brexpiprazole and cariprazine, and Q4 2019-Q3 2025 for lumateperone. Quarterly PS report counts were plotted descriptively for the available Q3 2020-Q3 2025 series and by post-approval quarter Q1-Q24 (Supplementary Figures S1-S2). These count plots lack exposure denominators and were not used to infer incidence, comparative risk, or a Weber effect.

### 2.4 Statistical analysis

For each target drug and outcome, OpenVigil generated a 2 × 2 table: a = reports containing both the target drug and event; b = reports containing the target drug without the event; c = reports containing the event without the target drug; and d = reports containing neither. Each drug was evaluated separately against the remaining database background; no formal pairwise comparison among the four drugs was performed.

The reporting odds ratio was ROR = (a/b)/(c/d), with 95% CI calculated on the log scale as exp[ln(ROR) ± 1.96√(1/a +1/b + 1/c + 1/d)]. The proportional reporting ratio was PRR = [a/(a+b)]/[c/(c + d)], accompanied by the Yates-corrected chi-square statistic. The Bayesian confidence propagation neural network measure was IC = log2{a(a+b+c+d)/[(a+b)(a+c)]}; IC025 denotes the lower 95% credibility bound (displayed by OpenVigil as “IC-2SD”) (Bate et al., 1998; Evans et al., 2001; van Puijenbroek et al., 2002).

Analyses required at least three reports. A frequentist signal was retained when the lower 95% ROR bound exceeded one and PRR was ≥2 with chi-square ≥4; IC025 > 0 provided supportive Bayesian evidence, and IC025 ≥ 1 was interpreted as a moderate-to-strong reporting signal. At PT level, 22 unique PTs were evaluated for each of four drugs (88 drug-PT evaluations). Because these analyses were exploratory signal detection, no family-wise or false-discovery-rate adjustment was applied. To make sparse-data uncertainty visible, retained PT signals with N < 20 were marked with † and treated as highly exploratory regardless of whether metric thresholds were met. Values were generated in OpenVigil 2.1 and tabulated in Microsoft Excel; no patient-level inferential modeling was performed.

Data-audit note: the archived SMQ/PT 2 × 2 outputs had target-drug margins (a+b) of 13,115 for lurasidone, 8,159 for brexpiprazole, 3,872 for cariprazine, and 2,254 for lumateperone. These margins differ from the descriptive PS-report totals in Table 1 (13,653; 10,661; 5,253; and 4,038). The retained extraction log does not document the reason for this difference. Signal estimates are therefore reproduced from the archived 2 × 2 cells and must not be assumed to represent the entire descriptive cohort; the discrepancy is an additional reproducibility limitation.

**TABLE 1:** Characteristics of primary-suspect reports for the four study drugs, Q1 2004–Q3 2025.

Characteristic	Lurasidone (%)	Brexpiprazole (%)	Cariprazine (%)	Lumateperone (%)
Number of reports	13,653	10,661	5,253	4,038
Gender				
Female	6,733 (49.32)	6,198 (58.14)	2,549 (48.52)	2,440 (60.43)
Male	3,032 (22.21)	2,857 (26.80)	1,314 (25.02)	1,130 (27.98)
Unknown	3,888 (28.47)	1,606 (15.06)	1,390 (26.46)	468 (11.59)
Age				
<18	430 (3.16)	254 (2.38)	225 (4.28)	14 (0.35)
18–64	3,228 (23.64)	2,597 (24.36)	1,145 (21.80)	1,517 (37.57)
65–80	435 (3.18)	816 (7.65)	177 (3.37)	128 (3.17)
>80	24 (0.18)	334 (3.14)	16 (0.30)	11 (0.27)
Unknown	9,536 (69.84)	6,660 (62.47)	3,690 (70.25)	2,368 (58.64)
Serious outcomes				
Death	278 (2.04)	530 (4.97)	137 (2.61)	33 (0.82)
Disability	101 (0.74)	52 (0.49)	44 (0.84)	13 (0.32)
Life-threatening	215 (1.57)	59 (0.55)	37 (0.70)	4 (0.10)
Hospitalization	1,308 (9.58)	775 (7.27)	364 (6.93)	258 (6.39)
Other	3,120 (22.85)	1818 (17.05)	702 (13.36)	698 (17.28)
Unknown	8,631 (63.22)	7,427 (69.67)	3,969 (75.56)	3,032 (75.09)
Reporter country				
United States	12,031 (88.12)	9,419 (88.35)	4,767 (90.75)	4,035 (99.93)
Japan	745 (5.46)	833 (7.81)	3 (0.06)	0 (0)
Canada	181 (1.32)	111 (1.04)	14 (0.27)	2 (0.05)
Australia	179 (1.31)	94 (0.88)	18 (0.34)	0 (0)
Other countries	517 (3.79)	204 (1.92)	451 (8.58)	1 (0.02)

Percentages use the drug-specific primary-suspect report total in the first row as denominator. Serious-outcome categories in the available aggregate output sum to the drug-specific total and were treated as mutually exclusive displayed categories.

## 3 Results

### 3.1 Descriptive analyses

The locked descriptive report summary contained 33,605 PS reports: 13,653 for lurasidone, 10,661 for brexpiprazole, 5,253 for cariprazine, and 4,038 for lumateperone. Approximate US post-approval observation intervals to the Q3 2025 cutoff were Q4 2010–Q3 2025, Q3 2015–Q3 2025, Q3 2015–Q3 2025, and Q4 2019–Q3 2025, respectively. Quarterly counts are shown in Figure 1 and demonstrate different accumulation patterns, including peaks for lurasidone in Q3 2017 and Q3 2021, brexpiprazole in Q3 2024, cariprazine in Q4 2020, and lumateperone in Q1 2024. These counts were not adjusted for prescriptions, patient exposure, market share, or calendar-time reporting changes. Table 1 uses the drug-specific descriptive PS-report total as the denominator. In the available aggregate output, the displayed serious-outcome categories sum to the drug-specific total and were treated as mutually exclusive categories.

**FIGURE 1:**
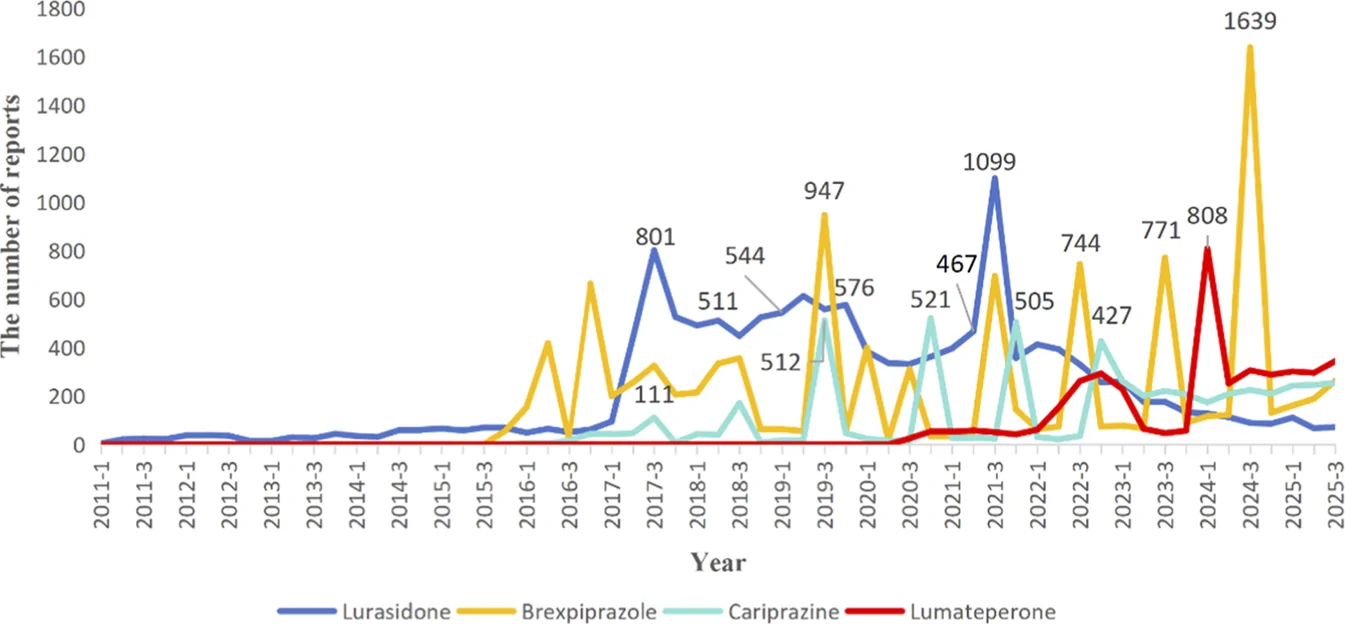
Quarterly primary-suspect report counts for the four study drugs through Q3 2025. Counts are descriptive and are not adjusted for prescriptions, exposed patients, market share, or changes in reporting over time.

Table 2 summarizes the most frequently recorded indications and concomitant medications. Percentages for indications used non-missing indication entries as denominators (lurasidone 13,115; brexpiprazole 8,160; cariprazine 3,872; lumateperone 2,254); percentages for concomitant medications used non-missing concomitant-medication entries (7,808; 6,077; 2,099; and 1,487, respectively). These variables were descriptive and were not used for confounding control. Supplementary Table S2 provides reporting context across all coded terms; product-use errors, off-label use, and product issues in that table are report terms and were not interpreted as clinical adverse reactions.

**TABLE 2:** Most frequently recorded indications and concomitant medications in primary-suspect reports.

Category	Lurasidone (n, %)	Brexpiprazole (n, %)	Cariprazine (n, %)	Lumateperone (n, %)
Indications	Bipolar disorder (2,585, 19.71)	Depression (2,670, 32.72)	Bipolar disorder (671, 17.33)	Bipolar disorder (872, 38.69)
	Schizophrenia (770, 5.87)	Adjuvant therapy (779, 9.55)	Depression (384, 9.92)	Schizophrenia (249, 11.05)
	Depression (400, 3.05)	Schizophrenia (617, 7.56)	Bipolar I disorder (379, 9.79)	Bipolar II disorder (135, 5.99)
	Bipolar I disorder (334, 2.55)	Bipolar disorder (331, 4.06)	Schizophrenia (221, 5.71)	Bipolar I disorder (89, 3.95)
Concomitant medication	Lamotrigine (406, 5.20)	Bupropion (299, 4.92)	Lamotrigine (127, 6.05)	Lamotrigine (109, 7.33)
	Quetiapine (334, 4.28)	Fluoxetine (239, 3.93)	Quetiapine (83, 3.95)	Lithium (73, 4.91)
	Lithium (318, 4.07)	Duloxetine (239, 3.93)	Clonazepam (63, 3.00)	Quetiapine (59, 3.97)
	Clonazepam (267, 3.42)	Clonazepam (215, 3.53)	Lithium (62, 2.95)	Bupropion (49, 3.30)
	Aripiprazole (232, 2.97)	Sertraline (209, 3.44)	Levothyroxine (47, 2.24)	Gabapentin (45, 3.03)

Indication percentages use non-missing indication entries as denominators (13,115; 8,160; 3,872; and 2,254). Concomitant-medication percentages use non-missing concomitant-medication entries (7,808; 6,077; 2,099; and 1,487). A report may include more than one indication or concomitant medication.

### 3.2 Disproportionality analyses at the SMQ level

All four drugs met the prespecified frequentist signal criteria for each selected narrow sub-SMQ (Table 3; Figure 2). Within each drug, Akathisia had the largest IC025: lurasidone 5.23, brexpiprazole 6.21, cariprazine 5.95, and lumateperone 4.63. Dyskinesia IC025 values were 3.77, 4.07, 4.24, and 3.24, respectively; Dystonia values were 3.32, 3.18, 3.19, and 2.05. Full ROR confidence intervals and the corresponding PRR, chi-square, IC, and IC025 values are presented in Table 3. Because each estimate used a separate drug-versus-database comparator, differences in numerical signal strength are descriptive and not pairwise tests.

**TABLE 3:** Drug-specific disproportionality estimates for selected narrow extrapyramidal sub-SMQs.

Drug	Narrow sub-SMQ	N	PRR (χ^2^)	ROR (95% CI)	IC (IC025)
Lurasidone	Akathisia	402	42.82 (15,821.29)	44.09 (39.86–48.77)	5.23 (5.08)
	Dyskinesia	678	14.04 (8,130.12)	14.72 (13.62–15.91)	3.77 (3.65)
	Dystonia	237	10.48 (2009.44)	10.65 (9.36–12.12)	3.32 (3.13)
Brexpiprazole	Akathisia	633	88.18 (51,539.39)	93.68 (86.27–101.74)	6.21 (6.08)
	Dyskinesia	654	17.34 (9,974.13)	18.40 (17.00–19.93)	4.07 (3.95)
	Dystonia	169	9.55 (1,279.46)	9.69 (8.32–11.28)	3.18 (2.95)
Cariprazine	Akathisia	292	80.09 (22,172.39)	84.75 (75.21–95.50)	5.95 (5.77)
	Dyskinesia	372	19.92 (6,647.98)	21.36 (19.22–23.75)	4.24 (4.08)
	Dystonia	88	10.07 (708.47)	10.22 (8.28–12.62)	3.19 (2.88)
Lumateperone	Akathisia	95	33.32 (2,924.12)	34.10 (27.8–41.83)	4.63 (4.33)
	Dyskinesia	145	10.06 (1,175.80)	10.40 (8.81–12.28)	3.24 (3.00)
	Dystonia	31	4.60 (83.93)	4.63 (3.25–6.60)	2.05 (1.54)

Abbreviations: SMQ, Standardised MedDRA, query; N, number of reports; PRR, proportional reporting ratio; ROR, reporting odds ratio; CI, confidence interval; IC, information component; IC025, lower 95% credibility bound (OpenVigil label: IC-2SD). Upper ROR, limits were reconstructed from the displayed rounded point estimate and lower limit using log-scale symmetry; minor rounding differences are possible. The archived 2 × 2 outputs had target-drug margins (a+b) of 13,115, 8,159, 3,872, and 2,254, which differ from the descriptive PS, totals in Table 1 for reasons not documented in the retained extraction log. Each drug was compared separately with the remaining database background; values are not head-to-head estimates.

**FIGURE 2:**
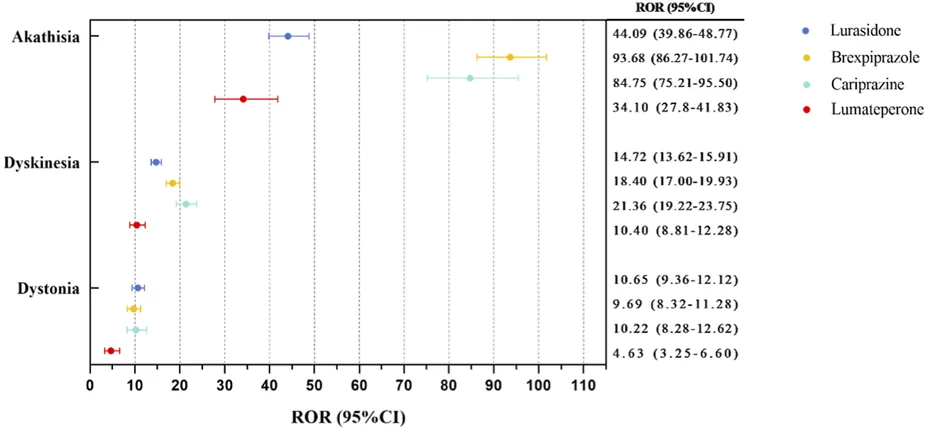
Drug-specific RORs for the narrow Akathisia, Dyskinesia, and Dystonia sub-SMQs. Each drug was compared separately with the remaining FAERS database background; the estimates are not formal head-to-head comparisons. The archived target-drug margins used in these estimates differed from the descriptive totals in Table 1 (see methods and Table 3 note).

### 3.3 Sensitivity analyses and longitudinal trends

All three selected sub-SMQs retained a lower 95% ROR confidence bound above 1 and IC025 above 0 for all four drugs in both sensitivity analyses (24 of 24 drug-outcome-window estimates; Supplementary Table S3). Signal magnitudes changed across windows, indicating that reporting disproportionality was time dependent. The persistence of positive estimates shows that signal detection was not confined to the full-period analysis, but it does not establish a stable cross-drug hierarchy or comparative risk. Quarterly report counts fluctuated and showed product-specific spikes rather than a uniform temporal pattern (Supplementary Figures S1-S2). Because these counts were not adjusted for utilization or patient exposure, the plots cannot distinguish changes in use from reporting artifacts or changes in event occurrence.

### 3.4 PT-level signal characterization

Across the three sub-SMQs, 22 unique PT labels were evaluated after removal of duplicate labels. Among PTs meeting the frequentist criteria, moderate-to-strong Bayesian signals (IC025 ≥ 1) numbered 17 for lurasidone, 10 for brexpiprazole, 12 for cariprazine, and four for lumateperone (Table 4). The number of detected PTs depends on report volume, coding, observation time, and the signal threshold and should not be interpreted as a comparative safety ranking. Of the 59 retained drug-PT signals shown in Table 4, 31 had N < 20 and are marked †; these sparse signals were considered highly exploratory.

**TABLE 4:** PT-level reporting signals within the selected narrow sub-SMQs.

Sub-SMQ / Preferred Term (PT)	N	PRR (χ^2^)	ROR (95% CI)	IC (IC025)
Akathisia				
Extrapyramidal disorder	110*	5.48 (396.69)	5.52 (4.57–6.67)	2.39 (2.11)
Movement disorder	58	2.75 (62.92)	2.76 (2.13–3.58)	1.42 (1.04)
Psychomotor hyperactivity	57*	5.57 (208.24)	5.59 (4.31–7.25)	2.36 (1.98)
Restlessness	438*	21.09 (8,213.60)	21.78 (19.78–23.98)	4.31 (4.17)
Dyskinesia				
Drooling	28*	7.43 (148.35)	7.45 (5.13–10.82)	2.60 (2.06)
Grimacing	6†	11.78 (48.38)	11.79 (5.27–26.38)	2.21 (1.11)
Muscle twitching	90	6.66 (424.99)	6.70 (5.44–8.25)	2.64 (2.34)
Oculogyric crisis	25*	16.19 (336.21)	16.22 (10.92–24.09)	3.34 (2.77)
Protrusion tongue	7†	19.16 (101.21)	19.17 (9.08–40.47)	2.54 (1.51)
Tardive dyskinesia	391*	16.16 (5,475.42)	16.63 (15.03–18.40)	3.94 (3.79)
Tic	42	11.70 (396.17)	11.73 (8.65–15.91)	3.22 (2.77)
Dystonia				
Blepharospasm	28	10.09 (218.28)	10.11 (6.97–14.66)	2.93 (2.39)
Facial spasm	10†	13.97 (106.40)	13.98 (7.49–26.09)	2.67 (1.80)
Laryngospasm	6†	4.15 (11.34)	4.15 (1.86–9.26)	1.51 (0.42)
Muscle contractions involuntary	6†	2.87 (5.52)	2.87 (1.29–6.39)	1.18 (0.08)
Muscle tightness	43	4.57 (115.87)	4.58 (3.39–6.19)	2.07 (1.64)
Musculoskeletal stiffness	112*	2.25 (76.51)	2.26 (1.86–2.75)	1.15 (0.88)
Oromandibular dystonia	17*†	40.50 (593.77)	40.55 (24.98–65.82)	3.65 (2.95)
Posture abnormal	7†	3.91 (12.35)	3.91 (1.86–8.22)	1.52 (0.49)
Tongue spasm	4*†	22.01 (59.36)	22.02 (8.18–59.28)	2.08 (0.77)
Torticollis	12*†	9.39 (81.08)	9.40 (5.32–16.61)	2.51 (1.70)
Trismus	50*	15.08 (634.60)	15.13 (11.44–20.01)	3.55 (3.14)

Panel B. Brexpiprazole — PT	N	PRR (χ^2^)	ROR (95% CI)	IC (IC025)
Akathisia				
Extrapyramidal disorder	89*	7.12 (460.80)	7.19 (5.83–8.87)	2.73 (2.43)
Movement disorder	—	—	—	—
Psychomotor hyperactivity	28*	4.39 (69.77)	4.40 (3.03–6.39)	1.97 (1.43)
Restlessness	162*	12.39 (1,675.39)	12.62 (10.80–14.75)	3.52 (3.30)
Dyskinesia				
Drooling	24	10.23 (189.56)	10.25 (6.86–15.32)	2.90 (2.32)
Grimacing	—	—	—	—
Muscle twitching	59	7.00 (296.82)	7.05 (5.45–9.12)	2.67 (2.29)
Oculogyric crisis	40	42.02 (1,524.51)	42.23 (30.84–57.83)	4.38 (3.92)
Protrusion tongue	—	—	—	—
Tardive dyskinesia	332*	22.02 (6,569.60)	22.91 (20.51–25.59)	4.36 (4.19)
Tic	18†	8.01 (103.14)	8.03 (5.05–12.77)	2.54 (1.88)
Dystonia				
Blepharospasm	15*†	8.65 (93.58)	8.67 (5.22–14.40)	2.54 (1.82)
Facial spasm	3†	6.68 (9.32)	6.68 (2.15–20.75)	1.46 (0.02)
Laryngospasm	—	—	—	—
Muscle contractions involuntary	—	—	—	—
Muscle tightness	13†	2.21 (7.48)	2.22 (1.29–3.82)	1.03 (0.25)
Musculoskeletal stiffness	—	—	—	—
Oromandibular dystonia	4†	14.90 (38.55)	14.90 (5.57–39.86)	1.98 (0.68)
Posture abnormal	—	—	—	—
Tongue spasm	—	—	—	—
Torticollis	5†	6.26 (17.09)	6.26 (2.60–15.07)	1.74 (0.55)
Trismus	14†	6.72 (62.36)	6.73 (3.98–11.38)	2.28 (1.53)

Panel C. Cariprazine — PT	N	PRR (χ^2^)	ROR (95% CI)	IC (IC025)
Akathisia				
Extrapyramidal disorder	80*	13.48 (910.12)	13.75 (11.01–17.17)	3.54 (3.22)
Movement disorder	20	3.21 (28.31)	3.22 (2.08–4.98)	1.54 (0.91)
Psychomotor hyperactivity	—	—	—	—
Restlessness	98*	15.75 (1,335.62)	16.14 (13.20–19.73)	3.77 (3.48)
Dyskinesia				
Drooling	18*†	16.14 (239.74)	16.21 (10.19–25.79)	3.16 (2.50)
Grimacing	—	—	—	—
Muscle twitching	30*	7.49 (162.18)	7.54 (5.27–10.79)	2.63 (2.11)
Oculogyric crisis	15*†	32.72 (426.37)	32.85 (19.74–54.67)	3.45 (2.73)
Protrusion tongue	5†	46.15 (175.81)	46.21 (19.12–111.68)	2.43 (1.25)
Tardive dyskinesia	192*	26.70 (4,698.17)	28.04 (24.24–32.44)	4.55 (4.34)
Tic	8†	7.49 (38.65)	7.50 (3.75–15.00)	2.12 (1.16)
Dystonia				
Blepharospasm	10†	12.14 (91.11)	12.17 (6.54–22.65)	2.59 (1.72)
Facial spasm	5†	23.52 (85.92)	23.55 (9.77–56.77)	2.30 (1.12)
Laryngospasm	—	—	—	—
Muscle contractions involuntary	—	—	—	—
Muscle tightness	11*†	3.95 (21.35)	3.96 (2.19–7.16)	1.66 (0.83)
Musculoskeletal stiffness	42*	2.85 (48.89)	2.87 (2.12–3.89)	1.45 (1.01)
Oromandibular dystonia	4*†	31.40 (88.53)	31.43 (11.74–84.14)	2.15 (0.85)
Posture abnormal	—	—	—	—
Tongue spasm	—	—	—	—
Torticollis	—	—	—	—
Trismus	10*†	10.11 (73.04)	10.13 (5.44–18.86)	2.47 (1.59)

Panel D. Lumateperone — PT	N	PRR (χ^2^)	ROR (95% CI)	IC (IC025)
Akathisia				
Extrapyramidal disorder	24*	6.93 (116.00)	7.00 (4.68–10.47)	2.48 (1.90)
Movement disorder	10*†	2.76 (9.53)	2.77 (1.49–5.15)	1.25 (0.38)
Psychomotor hyperactivity	—	—	—	—
Restlessness	19*†	5.23 (60.87)	5.27 (3.35–8.29)	2.11 (1.46)
Dyskinesia				
Drooling	4*†	6.14 (12.45)	6.15 (2.31–16.37)	1.60 (0.30)
Grimacing	—	—	—	—
Muscle twitching	19*†	8.15 (112.04)	8.21 (5.22–12.91)	2.58 (1.94)
Oculogyric crisis	—	—	—	—
Protrusion tongue	—	—	—	—
Tardive dyskinesia	62*	14.74 (779.97)	15.13 (11.75–19.48)	3.59 (3.22)
Tic	—	—	—	—
Dystonia				
Blepharospasm	—	—	—	—
Facial spasm	—	—	—	—
Laryngospasm	—	—	—	—
Muscle contractions involuntary	—	—	—	—
Muscle tightness	8*†	4.93 (21.29)	4.95 (2.47–9.92)	1.78 (0.81)
Musculoskeletal stiffness	19*†	2.22 (11.55)	2.23 (1.42–3.50)	1.06 (0.41)
Oromandibular dystonia	—	—	—	—
Posture abnormal	—	—	—	—
Tongue spasm	—	—	—	—
Torticollis	—	—	—	—
Trismus	—	—	—	—

PT, Preferred Term. “*” indicates that the exact PT, was explicitly named in, or included within an EPS, term grouping in, the current U.S., Prescribing Information available through DailyMed (DailyMed, 2025a; DailyMed, 2025b; DailyMed, 2026a; DailyMed, 2026b): LATUDA (revised January 2025), REXULTI (revised April 2026), VRAYLAR (revised December 2025), and CAPLYTA (revised April 2026); labels accessed 26 August 2026. “†” marks retained signals based on N < 20 reports and requiring particularly cautious interpretation. “—” indicates that no retained signal was present in the locked output. A report could contain multiple PTs. Upper ROR, limits were reconstructed from the displayed rounded point estimate and lower limit using log-scale symmetry; minor rounding differences are possible. Label inclusion and reporting signals do not establish incidence or causality.

The PT Extrapyramidal disorder was reported disproportionately for lurasidone [ROR 5.52 (95% CI 4.57–6.67)], brexpiprazole [7.19 (5.83–8.87)], cariprazine [13.75 (11.01–17.17)], and lumateperone [7.00 (4.68–10.47)]. Restlessness and Muscle twitching also met the frequentist criteria for all four drugs. Asterisks in Table 4 identify PTs explicitly named in, or included within an EPS term grouping in, the current U.S. Prescribing Information; label inclusion provides context and does not imply incidence or causality.

Additional retained PT signals included Grimacing, Laryngospasm, Muscle contractions involuntary, Posture abnormal, and Tongue spasm for lurasidone; Oculogyric crisis for lurasidone, brexpiprazole, and cariprazine; and Tardive dyskinesia for all four drugs. Several of these observations were supported by small counts, and isolated high disproportionality estimates were therefore not interpreted as robust between-drug differences. All PT findings remain hypothesis-generating and require clinical validation.

## 4 Discussion

### 4.1 Principal findings and interpretation

Using a consistent narrow-SMQ/PT framework, this study detected disproportionate reporting of Akathisia, Dyskinesia, and Dystonia for each of four newer SGAs. Akathisia was the most prominent selected category within each drug, while the PT analysis illustrated heterogeneity in reported movement-disorder terms. The results extend drug-specific signal characterization but do not show that one agent causes more or fewer events than another. Separate drug-versus-database RORs are influenced by each drug’s reporting environment and are not exchangeable estimates of comparative clinical risk.

### 4.2 Relation to previous evidence and pharmacology

Prior drug-specific FAERS studies have described movement-disorder reports for brexpiprazole, lumateperone, cariprazine, and lurasidone (Jiang et al., 2024; Zhao et al., 2024; Zhu et al., 2024; Luo et al., 2025). Randomized trials and meta-analyses likewise show that EPS liability can vary by drug and dose in selected trial populations (Huhn et al., 2019; Siafis et al., 2023). The incremental contribution of the present study is the application of the same selected narrow sub-SMQ and PT framework to four agents in one descriptive analysis, with explicit reporting of comparator definitions and uncertainty. This study is not a comparative safety assessment; label-listed events should be distinguished from signals that warrant further assessment.

Pharmacologic differences may help generate hypotheses but cannot explain an individual spontaneous report. Lurasidone is a D2/5-HT2A/5-HT7 antagonist with 5-HT1A partial agonism, whereas brexpiprazole and cariprazine are D2/D3 partial agonists with different intrinsic activity and receptor preferences; cariprazine also has long-lived active metabolites. Lumateperone has multimodal serotonergic, dopaminergic, and glutamatergic actions (Corponi et al., 2019; de Bartolomeis et al., 2022; Pahwa et al., 2021). Receptor occupancy, intrinsic activity, dose, active metabolites, prior treatment, and patient susceptibility may influence EPS expression, but these variables were not measured here; mechanistic interpretations therefore remain hypotheses.

### 4.3 Temporal and clinical sources of heterogeneity

The agents differed substantially in approval date, surveillance duration, cumulative use, and market penetration. Early post-marketing reporting, stimulated reporting after safety communications or label changes, regulatory attention, and secular changes in prescription volume may alter both report counts and disproportionality estimates. The common-calendar and product-age analyses preserved the direction of the selected signals but produced different magnitudes across windows (Supplementary Table S3). These findings reduce concern that signal positivity occurred only in the unequal full-period analysis, but they do not remove time-related reporting bias or support a relative safety hierarchy. The quarterly count plots showed product-specific fluctuations and spikes rather than a uniform early-peak pattern typical of the Weber effect (Hoffman et al., 2014) (Supplementary Figures S1-S2); without exposure denominators, these patterns cannot be interpreted as incidence or genuine drug-specific reporting propensity.

The descriptive indication and concomitant-medication distributions also differed across products. These variables may proxy disease severity, previous antipsychotic exposure, channeling, and centrally acting co-therapy. Restriction to PS reports reduces attribution ambiguity but does not control confounding. Because the retained files were aggregate, they could not support report-level indication stratification, exclusion of co-reported antipsychotics, or multivariable adjustment; each analysis requires a new case-level extraction.

### 4.4 Strengths and limitations

Strengths include the large spontaneous-reporting source, restriction to PS reports, consistent selected-outcome definitions, two time-window sensitivity analyses, and concordant use of frequentist and Bayesian signal measures. The limitations should be considered together. FAERS has no exposure denominator and is affected by under-reporting, over-reporting, selective and stimulated reporting, missing or inaccurate data, notoriety bias, and residual duplicates. MedDRA coding cannot substitute for clinical case validation, and temporality, dose, duration, dechallenge/rechallenge, and causality could not be adjudicated. Aggregate files did not permit report-level indication stratification, exclusion of co-reported antipsychotics, or multivariable adjustment. Parkinson-like events were not fully evaluated. The unexplained difference between descriptive PS totals and archived 2 × 2 target-drug margins limits reproducibility. In addition, 88 drug-PT evaluations were conducted without multiplicity adjustment, and 31 of 59 retained signals had N < 20. The common-calendar and product-age analyses explored sensitivity to surveillance time but could not remove time-related reporting bias or the absence of exposure denominators. These issues preclude causal inference and comparative safety ranking.

Future studies should retain report-level identifiers and dates, prespecify a common calendar window (e.g., Q1 2020–Q3 2025 for these four agents), include all four hierarchical EPS sub-SMQs, stratify by indication, exclude or model co-reported antipsychotics, evaluate demographic and reporter strata, and apply multiplicity-aware sensitivity analyses.

## 5 Conclusion

All four drugs showed disproportionate reporting for the selected Akathisia, Dyskinesia, and Dystonia sub-SMQs, with Akathisia the most prominent within-drug signal. The selected signals persisted in both time-window sensitivity analyses, although their magnitudes varied. Because Parkinson-like events were not fully evaluated, the analysis does not characterize the total EPS spectrum. The findings are hypothesis-generating and do not quantify incidence, comparative risk, or causal effects and should not guide preferential prescribing. Further validation requires case-level data to address indication, co-medication, and prior-treatment confounding and to complete EPS-domain coverage; exposure-adjusted or clinically adjudicated comparative data would be required for any risk comparison.

## Data Availability

The original contributions presented in the study are included in the article/Supplementary Material, further inquiries can be directed to the corresponding authors.
