# Supplementary material for: Reporting signals of selected extrapyramidal adverse events with four newer second-generation antipsychotics: a disproportionality analysis of FAERS spontaneous reports
Source: Front Pharmacol. 2026 Oct 12;17:1921943. doi: 10.3389/fphar.2026.1921943 (PMC13696228; doi:10.3389/fphar.2026.1921943)
Supplement: Supplementary file 1 [file DataSheet1.docx]

**Supplementary Table S1. Prespecified MedDRA v24 narrow sub-SMQs and included PT labels**

| **Narrow sub-SMQ** | **Code** | **Included PT labels** |
| --- | --- | --- |
| Akathisia | 20000096 | Extrapyramidal disorder; Movement disorder; Psychomotor hyperactivity; Restlessness |
| Dyskinesia | 20000097 | Drooling; Grimacing; Muscle twitching; Oculogyric crisis; Protrusion tongue; Tardive dyskinesia; Tic |
| Dystonia | 20000098 | Blepharospasm; Facial spasm; Laryngospasm; Muscle contractions involuntary; Muscle tightness; Musculoskeletal stiffness; Oromandibular dystonia; Posture abnormal; Tongue spasm; Torticollis; Trismus |

Note: These are the 22 unique PT labels evaluated in the locked aggregate analysis after duplicate labels were removed. Parkinson-like events (SMQ 20000099) were not available as a complete four-drug extraction and are outside the reported outcome set; therefore, this mapping represents selected rather than comprehensive EPS.

Supplementary Table S2. Most frequently reported terms across all organ systems (descriptive reporting context only)

| **Lurasidone** | **n** | **Brexpiprazole** | **n** | **Cariprazine** | **n** | **Lumateperone** | **n** |
| --- | --- | --- | --- | --- | --- | --- | --- |
| off label use | 1378 | weight increased | 1049 | off label use | 694 | dizziness | 289 |
| somnolence | 725 | product use in unapproved indication | 711 | akathisia | 225 | nausea | 227 |
| weight increased | 650 | akathisia | 531 | tardive dyskinesia | 192 | headache | 189 |
| insomnia | 622 | tardive dyskinesia | 332 | weight increased | 166 | somnolence | 143 |
| suicidal ideation | 516 | tremor | 278 | tremor | 139 | sedation | 108 |
| restlessness | 438 | incorrect dose administered | 225 | insomnia | 124 | burning sensation | 98 |
| mania | 423 | suicidal ideation | 169 | suicidal ideation | 107 | vomiting | 90 |
| tremor | 421 | restlessness | 162 | restlessness | 98 | mania | 83 |
| tardive dyskinesia | 391 | dyskinesia | 118 | extrapyramidal disorder | 80 | insomnia | 81 |
| akathisia | 380 | adverse event | 117 | vision blurred | 70 | dry mouth | 66 |
| dyskinesia | 267 | product administered to patient of inappropriate age | 106 | dyskinesia | 65 | suicidal ideation | 62 |
| suicide attempt | 165 | mania | 97 | musculoskeletal stiffness | 42 | tardive dyskinesia | 62 |
| withdrawal syndrome | 149 | drug effective for unapproved indication | 92 | dystonia | 30 | tremor | 49 |
| dystonia | 141 | extrapyramidal disorder | 89 | muscle twitching | 30 | akathisia | 46 |
| sedation | 136 | product prescribing error | 73 | restless legs syndrome | 27 | skin burning sensation | 45 |
| restless legs syndrome | 128 | therapeutic response unexpected | 67 | parkinsonism | 25 | migraine | 43 |
| completed suicide | 114 | sedation | 66 | sedation | 23 | paraesthesia | 41 |
| musculoskeletal stiffness | 112 | underdose | 66 | urinary retention | 23 | feeling hot | 39 |
| extrapyramidal disorder | 110 | increased appetite | 64 | movement disorder | 20 | chills | 35 |
| hypersomnia | 102 | dystonia | 61 | nightmare | 19 | hypersomnia | 32 |

Note: This supplementary table provides reporting context only. Product-use errors, off-label use, unapproved indications, and other product-use terms are coded report terms and are not interpreted as clinical adverse reactions.

Supplementary Table S3. Sensitivity Analyses for Selected Narrow Sub-SMQs: Common Calendar Window (Q1 2020–Q3 2025) vs. Product-Age Aligned Window (First 24 Quarters Post-Approval)

| Drug | Sub-SMQ | Full Period (Q1 2004–Q3 2025) | | Common Calendar Window (Q1 2020–Q3 2025) | | Product-Age Aligned Window (First 24 Quarters) | |
| --- | --- | --- | --- | --- | --- | --- | --- |
|  |  | ROR (95% CI) | IC (IC025) | ROR (95% CI) | IC (IC025) | ROR (95% CI) | IC (IC025) |
| Lurasidone | Akathisia | 44.09 (39.86-48.77) | 5.23 (5.08) | 44.40 (37.16-53.06) | 4.99 (4.73) | 15.43 (9.40-25.31) | 3.05 (2.34) |
|  | Dyskinesia | 14.72 (13.62-15.91) | 3.77 (3.65) | 26.51 (23.53-29.88) | 4.52 (4.34) | 4.80 (3.42-6.73) | 2.08 (1.59) |
|  | Dystonia | 10.65 (9.36-12.12) | 3.32 (3.13) | 19.15 (15.67-23.39) | 3.98 (3.69) | 7.43 (5.03-10.97) | 2.56 (1.99) |
| Brexpiprazole | Akathisia | 93.68 (86.27-101.74) | 6.21 (6.08) | 105.05 (92.82-118.90) | 6.13 (5.95) | 139.72 (125.02-156.15) | 6.47 (6.31) |
|  | Dyskinesia | 18.40 (17.00-19.93) | 4.07 (3.95) | 41.89 (37.96-46.22) | 5.12 (4.97) | 22.76 (19.96-25.95) | 4.30 (4.11) |
|  | Dystonia | 9.69 (8.32-11.28) | 3.18 (2.95) | 19.41 (15.92-23.66) | 4.00 (3.71) | 13.41 (10.63-16.92) | 3.49 (3.15) |
| Cariprazine | Akathisia | 84.75 (75.21-95.50) | 5.95 (5.77) | 112.44 (97.63-129.49) | 6.10 (5.90) | 104.66 (85.54-128.07) | 5.65 (5.35) |
|  | Dyskinesia | 21.36 (19.22-23.75) | 4.24 (4.08) | 43.10 (38.39-48.39) | 5.12 (4.95) | 21.81 (17.37-27.38) | 4.05 (3.72) |
|  | Dystonia | 10.22 (8.28-12.62) | 3.19 (2.88) | 18.37 (14.41-23.41) | 3.84 (3.49) | 15.18 (10.44-22.06) | 3.33 (2.79) |
| Lumateperone | Akathisia | 34.10 (27.8-41.83) | 4.63 (4.33) | 47.09 (38.32-57.87) | 4.94 (4.64) | 45.43 (36.98-55.81) | 4.91 (4.61) |
|  | Dyskinesia | 10.40 (8.81-12.28) | 3.24 (3.00) | 18.76 (15.88-22.16) | 4.01 (3.76) | 18.54 (15.70-21.90) | 3.99 (3.75) |
|  | Dystonia | 4.63 (3.25-6.60) | 2.05 (1.54) | 8.46 (5.49-12.06) | 2.77 (2.25) | 8.13 (5.71-11.59) | 2.72 (2.21) |

The number of reports

| Year | Lurasidone | Brexpiprazole | Cariprazine | Lumateperone |
| --- | --- | --- | --- | --- |
| 2020-3 | 332 | 312 | 17 | 25 |
| 2020-4 | 362 | 34 | 521 | 54 |
| 2021-1 | 396 | 36 | 27 | 53 |
| 2021-2 | 467 | 52 | 29 | 58 |
| 2021-3 | 1099 | 695 | 24 | 51 |
| 2021-4 | 357 | 144 | 505 | 42 |
| 2022-1 | 413 | 64 | 31 | 61 |
| 2022-2 | 393 | 74 | 22 | 152 |
| 2022-3 | 330 | 744 | 35 | 261 |
| 2022-4 | 256 | 74 | 427 | 292 |
| 2023-1 | 259 | 78 | 261 | 228 |
| 2023-2 | 175 | 67 | 200 | 65 |
| 2023-3 | 175 | 771 | 220 | 47 |
| 2023-4 | 133 | 90 | 206 | 57 |
| 2024-1 | 127 | 117 | 173 | 808 |
| 2024-2 | 113 | 122 | 208 | 251 |
| 2024-3 | 89 | 1639 | 224 | 306 |
| 2024-4 | 86 | 131 | 209 | 287 |
| 2025-1 | 111 | 160 | 242 | 301 |
| 2025-2 | 67 | 188 | 245 | 295 |
| 2025-3 | 72 | 262 | 253 | 344 |

Supplementary Figure S1. Quarterly primary-suspect report counts for the four study drugs through Q3 2020 - Q3 2025.

The number of reports

| Year | Lurasidone | Brexpiprazole | Cariprazine | Lumateperone |
| --- | --- | --- | --- | --- |
| Q1 | 5 | 1 | 0 | 0 |
| Q2 | 21 | 57 | 0 | 0 |
| Q3 | 25 | 154 | 6 | 0 |
| Q4 | 22 | 418 | 4 | 25 |
| Q5 | 38 | 24 | 17 | 54 |
| Q6 | 39 | 664 | 44 | 53 |
| Q7 | 36 | 199 | 42 | 58 |
| Q8 | 15 | 255 | 47 | 51 |
| Q9 | 15 | 325 | 111 | 42 |
| Q10 | 30 | 205 | 7 | 61 |
| Q11 | 26 | 214 | 43 | 152 |
| Q12 | 44 | 334 | 40 | 261 |
| Q13 | 35 | 356 | 170 | 292 |
| Q14 | 31 | 63 | 9 | 228 |
| Q15 | 59 | 63 | 17 | 65 |
| Q16 | 61 | 55 | 18 | 47 |
| Q17 | 66 | 947 | 512 | 57 |
| Q18 | 58 | 53 | 47 | 808 |
| Q19 | 70 | 400 | 24 | 251 |
| Q20 | 68 | 20 | 16 | 306 |
| Q21 | 49 | 312 | 17 | 287 |
| Q22 | 65 | 34 | 521 | 301 |
| Q23 | 51 | 36 | 27 | 295 |
| Q24 | 62 | 52 | 29 | 344 |

Supplementary Figure S2. The number of initial suspected case reports for four drugs, aligned according to the product age after launch (Q1-Q24).
